# Mindfulness, perceived social support, and suicidal ideation among Chinese adolescents: the mediating role of self-compassion

**DOI:** 10.3389/fpsyt.2025.1613442

**Published:** 2025-09-11

**Authors:** Shining Zhang, Yanzhen Ren, Haiping Zeng, Donghuan Rong, Hanlin Ren, Tingyun Jiang, Yuan Fang

**Affiliations:** Zhongshan Mental Health Center, The Third People’s Hospital of Zhongshan, Zhongshan, Guangdong, China

**Keywords:** mindfulness, perceived social support, self-compassion, suicidal ideation, adolescents

## Abstract

**Objective:**

Previous studies have demonstrated that mindfulness and perceived social support are protective factors against suicide. However, the potential common mechanisms underlying these relationships remain unclear. This study aimed to examine the associations between mindfulness, perceived social support, and suicidal ideation, and to explore the mediating role of self-compassion in these relationships among a sample of adolescents in China.

**Methods:**

A total of 1709 adolescents (M_age= 15.06, SD_age= 1.51) were recruited from one junior high school and one senior high school in Zhongshan, China. Mindfulness, perceived social support, suicidal ideation and self-compassion were assessed using self-report questionnaires. Structural Equation Modeling (SEM) was used to estimate the relationships between the variables.

**Results:**

Mindfulness and perceived social support were both positively associated with self-compassion, which in turn was negatively associated with suicidal ideation. Moreover, self-compassion partially mediated the relationship between mindfulness and suicidal ideation (indirect effect = -0.12, 95% confidence interval [CI] [-0.15, -0.08]), as well as the relationship between perceived social support and suicidal ideation (indirect effect = -0.08, 95% CI [-0.11, -0.06]).

**Conclusions:**

Mindfulness and perceived social support were associated with lower levels of suicidal ideation, and these associations were partially explained by self-compassion, suggesting that self-compassion may act as a shared mediator. Interventions that strengthen mindfulness, perceived social support, and self-compassion—such as mindfulness-based supportive group interventions or compassion-focused therapy—may be particularly beneficial for adolescents experiencing suicidal ideation.

## 1 Introduction

Suicide is a significant global public health concern, with more than 700,000 people dying by suicide each year (1). A survey has shown that suicide is the fourth leading cause of death among young adults aged 15 to 29 worldwide (1). Adolescents may be at particular risk for suicide owing to the multiple and rapid biological, psychological, and social changes that occur during this developmental stage (2). In recent years, the incidence of suicide-related behaviors among adolescents has increased significantly (3). In China, the prevalence of suicidal ideation among adolescents ranges from 10.7% to 24.2% (4). Given the high prevalence and serious consequences of adolescent suicide, it is crucial to investigate influencing factors and underlying mechanisms to prevent it.

According to the integrated motivation-volition model of suicidal behavior (5), self-attitudes play a significant role in the formation and development of suicide. Researchers posit that suicide represents an extreme form of self-criticism and self-attack (6), which contrasts with self-compassion. Self-compassion is characterized by kindness and compassion towards oneself in face of sufferings and failures (7). According to Neff (7), self-compassion comprises three main components: (1) self-kindness, an attitude of kindness and understanding towards oneself; (2) common humanity, the tendency to view one’s experiences as part of the broader human experience; (3) mindfulness, a balanced awareness of painful thoughts and experiences without over-identifying with them. Notably, the mindfulness component within self-compassion is related to but distinct from the broader construct of mindfulness, the latter encompasses additional abilities such as attention, non-judgment and openness (8, 9). Previous studies have found that self-compassion is a crucial protective factor against suicide (10, 11). For example, a cross-sectional study of 4873 Chinese adults found that self-compassion is associated with reduced suicide risk (12). Similar findings have also emerged in studies of veterans, youth, and victims of intimate partner abuse (13–15). Neuroimaging work also suggests potential neural pathways linking self-compassion and suicidal ideation—for instance, associations have been reported between self-compassion and self-referential caudate circuitry implicated in adolescent suicidal thoughts (16). Moreover, emerging intervention studies provide preliminary evidence that mindful self-compassion programs can reduce suicidal ideation in adolescent samples, underscoring the translational potential of targeting self-compassion in prevention efforts (17). Given the prevalence of self-criticism and high self-expectations in traditional Chinese culture, promoting self-compassion among adolescents may be particularly important for suicide prevention in China.

The cultivation and enhancement of self-compassion, on the one hand, is the result of the internal factors such as mindfulness (18); On the other hand, it is also influenced by perceived external social support (19). Mindfulness is defined as the state of attention and awareness of the present moment with an attitude of nonjudgemental (20, 21). When confronted with internal suffering, a state of mindful awareness allows individuals to acknowledge their pain without judgment, subsequently fostering feelings of self-kindness and common humanity to soothe themselves (22). Consistent with these views, a growing body of studies suggest that mindfulness promotes self-compassion and that self-compassion is one of the potential mechanisms through which mindfulness affects mental health (23, 24). Cross-sectional studies have found associations between mindfulness and higher self-compassion, and longitudinal studies indicate that mindfulness predicts subsequent increases in self-compassion (25, 26). Moreover, numerous studies examining the effect of mindfulness-based training programs have shown an elevation in self-compassion (27, 28).

Perceived social support refers to the perception that one is cared for by others and has a reliable social network to turn to in times of need (29). Individuals may perceive social support from various sources, including family, friends, and significant others (29). According to the integrative model of social support proposed by Feeney and Collins (30), social support can influence an individual’s internal self-attitudes, such as self-compassion (31, 32). Neff also argued that self-compassion may be developed and influenced by the important relationships with others (8). Supporting this view, numerous studies have found a positive correlation between perceived social support and self-compassion (33–35).

Previous theories and studies have demonstrated that suicide is not only influenced by internal factors (e.g., mindfulness) but also closely related to external factors (e.g., perceived social support) (36). On the one hand, mindfulness has been inversely associated with suicidal behavior, and mindfulness-based interventions could reduce individuals’ suicide risk (37–39). On the other hand, perceived social support shows a strong negative association with suicide ideation, and a lack of social support increases the risk of suicide (40, 41).

In summary, mindfulness and perceived social support have protective effects against suicide, with self-compassion potentially acting as a mediator in these relationships. However, few studies have explored these four variables in combination. To facilitate understanding of the relationships among these variables and to test whether mindfulness and perceived social support operate through shared pathways, the present study examined whether self-compassion mediates the relationships of mindfulness and perceived social support with suicidal ideation by conducting a survey among adolescents in China. In addition, we examined whether mindfulness and perceived social support interact in predicting suicidal ideation. Testing this interaction allows us to determine whether the protective effect of mindfulness varies across levels of perceived social support (or vice versa), thereby identifying subgroups for which mindfulness-based or social-support interventions may be particularly useful. Such an examination is theoretically grounded in stress-buffering and resilience frameworks, which posit that internal resources (e.g., mindfulness) and external resources (e.g., social support) may jointly and conditionally influence risk for suicidal ideation. The findings may provide preliminary insights into mechanisms linking these factors and could inform the design of future intervention studies (e.g., mindfulness-based supportive group programs) aimed at prevention.

## 2 Method

### 2.1 Participants

This cross-sectional study was conducted in one junior high school and one senior high school in Zhongshan City, Guangdong Province, China. The schools were selected via convenience sampling, and all students at the selected schools were invited to participate. A total of 1973 adolescents were recruited between February and June 2023. Participants with missing or internally inconsistent responses were excluded from analyses. The final sample comprised data from 1709 adolescents (effective response rate: 86.62%). Participants were aged 12–18 years (Mean_age=15.16 years, SD=1.51); 873(51.1%) were male, and 836 (48.9%) were female.

### 2.2 Procedure

Prior to beginning the survey, participants were informed about the study’s aims, the confidentiality of their responses, and their right to withdraw at any time. Informed consent was obtained from all participants. Subsequently, participants completed a series of paper-and-pen questionnaires in quiet school classrooms at a prescribed time. This study protocol was approved by the Ethics Committee of The Third People’s Hospital of Zhongshan.

### 2.3 Measures

#### 2.3.1 Mindful attention awareness scale

Mindfulness was measured using the Mindful Attention Awareness Scale (MAAS) (20), a 15-item self-report instrument that captures mindfulness in its core aspects of attention and awareness. Items in this scale are rated on a 6-point Likert scale ranging from 1(almost always) to 6 (almost never), with higher scores indicating higher levels of mindfulness. The MAAS has demonstrated good reliability in Chinese samples (42). In the present study, the scale also showed good internal reliability (Cronbach’s alpha = 0.91).

#### 2.3.2 Multidimensional scale of perceived social support

Perceived social support was assessed using the Chinese revision of the Multidimensional Scale of Perceived Social Support (MSPSS) (43, 44). This 12-item instrument measures perceived social support from family, friends, and significant others. Items are rated on a 7-point Likert scale (1 = very strongly disagree to 7 = very strongly agree). Higher scores indicate higher levels of perceived social support. The MSPSS has demonstrated strong validity and reliability in the Chinese population (43, 45). In this study, the MSPSS also displayed good internal consistency (Cronbach’s alpha = 0.94).

#### 2.3.3 Self-compassion scale

Self-compassion was measured with the Chinese revision of the Self-Compassion Scale (SCS) (8, 46). The Chinese short form of SCS contains 12 items assessing three dimensions of self-compassion: self-kindness, common humanity, and mindfulness. Items are rated on a 5-point Likert scale (e.g., 1 = almost never to 5 = almost always). After appropriate recoding, higher scores represent greater levels of self-compassion. The SCS has demonstrated excellent validity and reliability among Chinese adolescents (46). In the present study, the SCS also displayed good internal consistency (Cronbach’s alpha = 0.85).

#### 2.3.4 Item 9 of the beck depression inventory-II

Suicide ideation was assessed using item 9 of the Beck Depression Inventory-II (BDI-II), which asks about suicidal thoughts or wishes over the past two weeks (47). Item 9 is scored from 0 to 3: 0 = “I don’t have any thoughts of killing myself”; 1 = “I have thoughts of killing myself, but I would not carry them out”; 2 = “I would like to kill myself”; 3 = “I would kill myself if I had the chance.” Higher scores indicate more severe suicidal ideation.

### 2.4 Data analyses

First, preliminary analyses involved descriptive statistics and correlations performed using SPSS 20.0. Structural Equation Modeling (SEM) was then conducted using AMOS 24.0 to test the mediating effects of self-compassion. The model included mindfulness and perceived social support as independent variables, suicidal ideation as the dependent variable, and self-compassion as the mediator. Prior to model estimation, all variables were standardized to reduce multicollinearity. In addition to testing direct and mediation paths, we included an interaction term between mindfulness and perceived social support to examine whether the protective association of mindfulness with suicidal ideation varied by level of perceived social support. Model fit was evaluated using the following indices (48): (1) the ratio of chi-square to degrees of freedom (χ^2^/df); (2) the comparative fit index (CFI), with values > 0.90 indicating adequate fit; (3) the Tucker-Lewis index (TLI), with values > 0.90 indicating adequate fit; (4) the standardized root mean square residual (SRMR), with values < 0.08 indicating acceptable fit; and (5) the root mean square error of approximation (RMSEA), with values < 0.08 indicating acceptable fit. Because the chi-square statistic is sensitive to large sample sizes, greater emphasis was placed on incremental and absolute fit indices (CFI, TLI, SRMR, RMSEA) when evaluating model adequacy. Finally, a bias-corrected bootstrapping procedure (with 5000 resamples) was employed to test the significance of the mediation model. A significance level of 0.05 was used for all statistical tests in this study.

## 3 Results

As shown in **Table 1**, regarding suicidal ideation, 1379 participants (80.7%) reported that they didn’t have any thoughts of killing themselves; 295 participants (17.3%) reported that they had thoughts of killing themselves, but would not carry out; 26 participants (1.5%) reported that they would like to kill themselves; and 9 participants (0.5%) reported that they would kill themselves if they had the chance. Overall, approximately 19.3% of participants exhibited some degree of suicidal ideation.

**Table 1:** Demographics and clinical feature of participants (N=1709).

Variables	Contents	*n (%) or (M ± SD)*
Age		15.16±0.86
Sex	Male	873 (51.1%)
	Female	836 (48.9%)
Suicidal ideation	0 I don’t have any thoughts of killing myself	1379 (80.7%)
	1 I have thoughts of killing myself, but I would not carry them out	295 (17.3%)
	2 I would like to kill myself	26 (1.5%)
	3 I would kill myself if I had the chance	9 (0.5%)

*M±SD*, Mean and standard deviation.

The means, standard deviations (SDs), and bivariate correlations for all variables are presented in **Table 2**. Suicidal ideation was negatively correlated with mindfulness (r= -0.31; p< 0.01), perceived social support (r= -0.31; p< 0.01) and self-compassion (r= -0.37; p< 0.01). In contrast, self-compassion was positively correlated with mindfulness (r= 0.61; p< 0.01) and perceived social support (r= 0.53; p< 0.01).

**Table 2:** Pearson’s r correlations between the variables.

Variables	M(SD)	(2)	(3)	(4)
(1) Mindfulness	60.93 (13.89)	0.45^**^	0.61^**^	-0.31^**^
(2) Perceived social support	62.51 (14.15)		0.53^**^	-0.31^**^
(3) Self-compassion	40.60 (8.64)			-0.37^**^
(4) Suicidal ideation	0.22 (0.48)			

^*^ *p* < 0.05; ^**^ *p* < 0.01.

**Figure 1:**
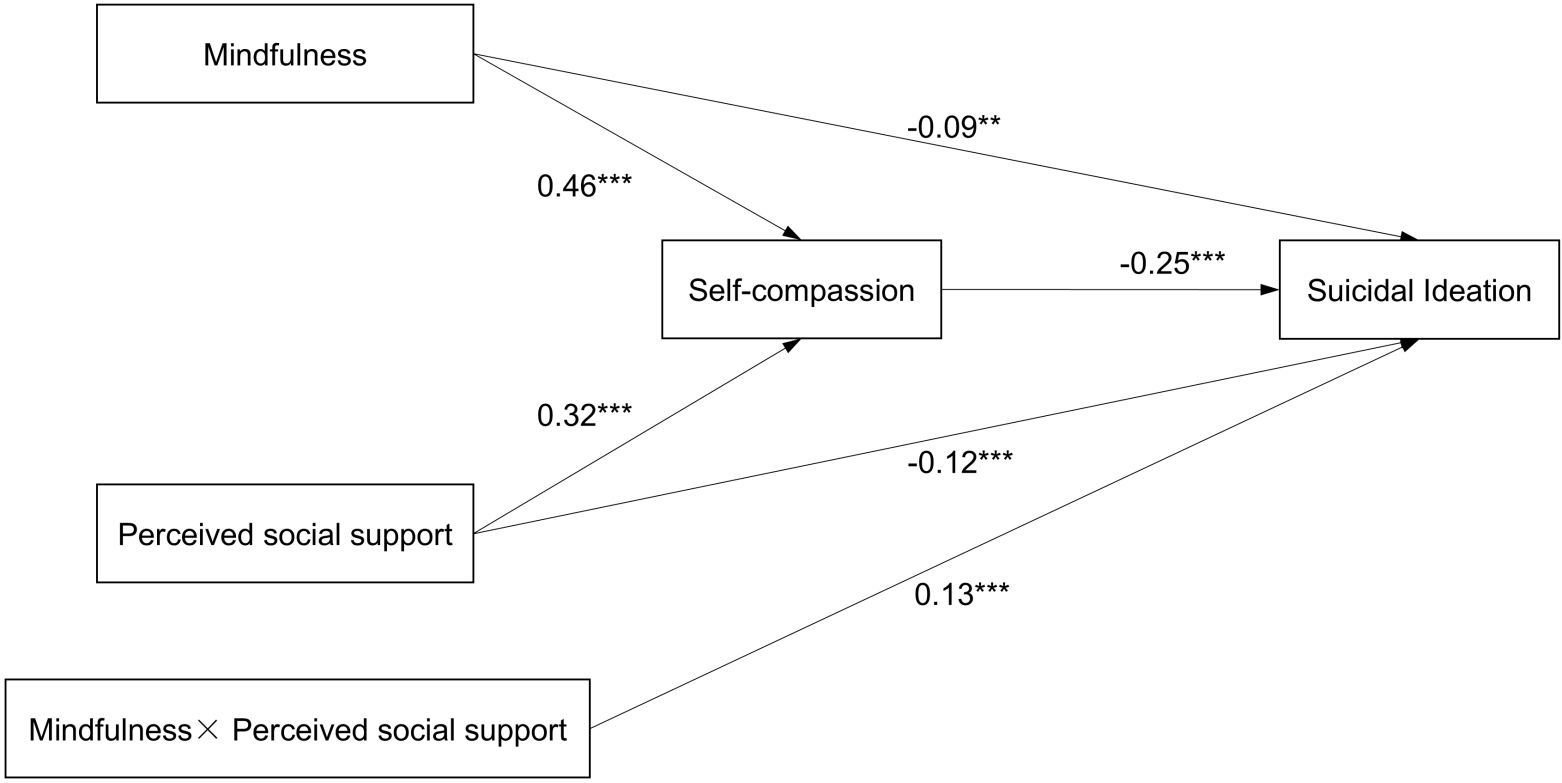
Path diagram with structural equation modeling results and standardized path coefficients. *p < 0.05; **p < 0.01; ***p < 0.001.

The SEM model with standardized beta coefficients is presented in **Figure 1**. The hypothesized model demonstrated a good fit with the data (χ^2^/df=7.757, p=0.005, CFI=0.996, TLI=0.962, SRMR=0.013, RMSEA=0.063). Although the χ^2^ statistic was significant and the χ^2^/df was relatively large, this is not unexpected given the large sample size (n = 1709) and the sensitivity of χ^2^ to sample size; therefore, model adequacy was interpreted primarily on the basis of CFI, TLI, SRMR and RMSEA. CFI and TLI both exceeded commonly used thresholds for good fit, SRMR was well below 0.08, and RMSEA fell within the acceptable range, together indicating that the specified model fit the data well. All pathways in the model were significant (ps<0.01). Specifically, both mindfulness and perceived social support positively predicted self-compassion (β= 0.46, β= 0.32, respectively; ps< 0.01), which in turn negatively predicted suicidal ideation (β= -0.25, p< 0.01). In addition, the direct paths from mindfulness and perceived social support to suicidal ideation remained significant while controlling for self-compassion (β= -0.09, β= -0.12, respectively; ps< 0.01), indicating that self-compassion partially mediated these relationships. Furthermore, bootstrap analyses demonstrated that self-compassion mediated the relationship between mindfulness and suicidal ideation (indirect effect= -0.12, 95% CI [-0.15, -0.08]) as well as the relationship between perceived social support and suicidal ideation (indirect effect= -0.08, 95% CI [-0.11, -0.06]) (as shown in **Table 3**).

**Table 3:** The paths and effect analysis.

Effect	Paths	Effect size	95% CI
Direct effect	Mindfulness→ Suicidal ideation	-0.09	-0.15- -0.03
Indirect effect	Mindfulness→ Self-compassion →Suicidal ideation	-0.12	-0.15- -0.08
Direct effect	Perceived social support→ Suicidal ideation	-0.12	-0.17- -0.06
Indirect effect	Perceived social support→ Self-compassion →Suicidal ideation	-0.08	-0.11- -0.06

The study identified a significant interaction between mindfulness and perceived social support in predicting suicidal ideation (β= 0.13, p< 0.01). A simple slope analysis was conducted to further investigate this interaction by assessing the effects of mindfulness on suicidal ideation at high (+1SD) and low (-1SD) levels of perceived social support. The results indicated that the negative association between mindfulness and suicidal ideation was significant at both low (simple slope = -0.41, p< 0.01) and high (simple slope = -0.23, p< 0.01) levels of perceived social support.

**Figure 2:**
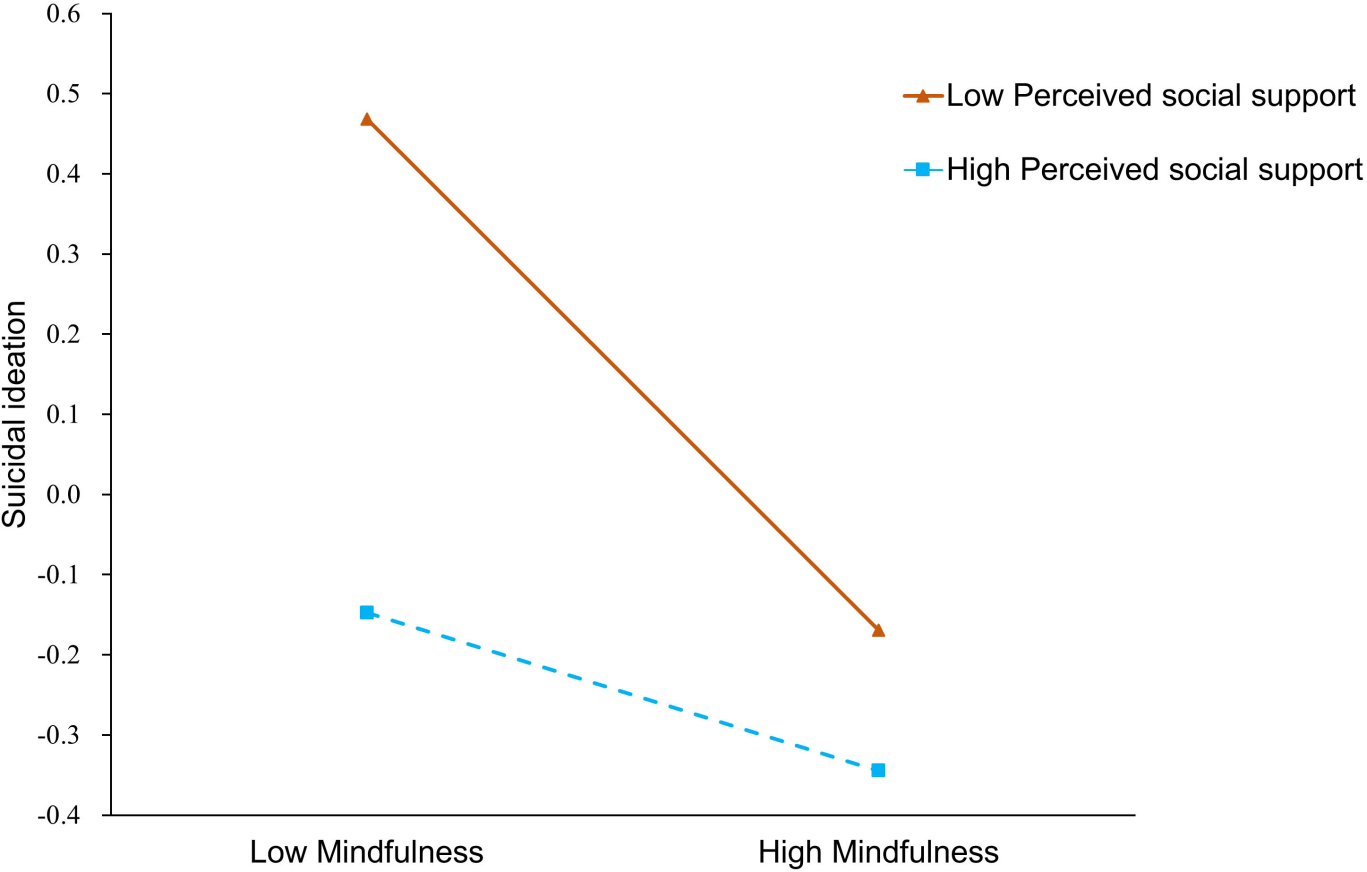
Moderation effect of perceived social support on the relation between mindfulness and suicidal ideation. High and low values are 1 standard deviation above and below the mean, respectively.

While both groups those with low and high perceived social support- exhibited elevated levels of suicidal ideation when mindfulness was low, however the slope was notably steeper for individuals with low perceived social support, indicating a stronger negative relationship as levels of perceived social support decreased. In other words, perceived social support appeared to mitigate the high suicidal ideation associated with low mindfulness (see **Figure 2**).

## 4 Discussion

Traditional Chinese culture, profoundly shaped by Confucianism, emphasizes continuous self-improvement and critical self-criticism (e.g., “being strict with oneself, lenient toward others” and “examining oneself thrice daily”) (49, 50). While this cultural tradition facilitates personal growth and social harmony, it may conflict with the core principle of self-compassion—treating oneself kindly in a non-judgmental way (51). This cultural tension may foster harsh self-criticism and psychological stress, making it more difficult for Chinese adolescents to treat themselves with kindness. For example, when facing academic setbacks or interpersonal conflicts, Chinese parents are more likely to encourage their children to identify their own shortcomings rather than provide emotional support (52, 53). In more extreme cases, even when children experience unjust treatment, parents may still encourage self-reflection. Such practices can socialize children into a harshly critical stance toward themselves rather than toward self-compassion (54). Consequently, in times of failure and distress, adolescents may be more inclined to cope by engaging in self-harming behaviors (55). Therefore, understanding the mechanisms through which mindfulness and perceived social support influence suicide risk—and the potentially central mediating role of self-compassion—is important for developing targeted interventions to reduce suicide risk among Chinese adolescents.

While previous studies have examined mindfulness and perceived social support in relation to suicidal ideation in other populations, the present study is among the first to investigate self-compassion as a potential common mechanism linking these protective factors to suicidal ideation specifically among Chinese adolescents. The main findings are: (1) high levels of mindfulness and perceived social support are negatively associated with suicidal ideation; (2) self-compassion partially mediates the associations between mindfulness and suicidal ideation, as well as between perceived social support and suicidal ideation; (3) mindfulness and perceived social support have an interactive effect on suicidal ideation.

The present study found that mindfulness and perceived social support are negatively associated with suicidal ideation, which is consistent with prior research (39–41, 56). Specifically, individuals with higher levels of mindfulness and perceived social support have lower levels of suicidal ideation. On the one hand, according to the escape theory of suicide, suicide is a way to escape emotional pain (57), while mindfulness emphasizes acceptance of present experiences, whether pleasant or painful, so individuals with high levels of mindfulness are less likely to have suicidal ideations (58). On the other hand, the interpersonal theory of suicide points out that suicide desire arises from two frustrated interpersonal states: perceived burdensomeness and thwarted belongingness (59). High levels of perceived social support can reduce feelings of thwarted belongingness and perceived burdensomeness, thereby lowering the risk of suicidal ideation (60, 61).

As expected, the present study found that self-compassion partially mediates the effects of mindfulness and perceived social support on suicidal ideation, suggesting that self-compassionate attitudes can be fostered by mindfulness and social support and, in turn, have a protective effect on an individual’s suicidal ideation.

Mindfulness is generally considered to be an important factor in promoting self-compassion, and self-compassion is one of the potential mechanisms through which mindfulness influences mental health (26, 62). A state of mindful awareness when facing suffering or failure allows individuals to acknowledge and accept their thoughts, feelings, and bodily sensations in the present moment in a nonjudgemental manner (18). This specific relationship with experiences facilitates individuals adopting a gentler, kinder attitude toward themselves (63). According to Neff and Dahm (22), mindful awareness of suffering is a necessary prerequisite to compassionate responding.

Individuals who perceive higher levels of social support are more likely to develop self-compassion (33, 34). Germer has argued that people’s attitudes toward themselves are shaped by the evaluations of significant others (64), indicating that social support from important others plays a crucial role in fostering a compassionate self-attitude (65). On the one hand, when individuals perceive adequate support, care, and kindness from significant others, they are more likely to view themselves as worthy of care and to treat themselves with kindness and compassion (33). On the other hand, when individuals face adversities, if significant others understand individuals’ flaws and difficulties and respond with warmth, individuals are more likely to forgive their own failures or shortcomings, which can increase self-compassion (65, 66). Supporting this, Neff and McGehee reported that adolescents who received greater maternal praise and support exhibited higher levels of self-compassion compared to those who experienced stressful family relationships and frequent maternal criticism (66).

In summary, mindfulness and perceived social support promote self-compassion, which in turn may protect against suicidal ideation. Suicide can be seen as an extreme manifestation of negative self-directed attitudes (e.g., self-criticism, self-blame, self-loathing, self-attack) (11). Self-compassion fosters kindness and tolerance toward oneself, potentially alleviating these negative attitudes, and thus reducing suicidal ideation (11). Prior studies have found that self-compassion protects against suicidality by alleviating negative internal experiences such as self-blame (14). In addition, the interpersonal theory of suicide (59) suggests that suicidal ideation often arises when individuals perceive themselves as a burden to others (perceived burdensomeness) and lack a sense of belonging (thwarted belongingness). Self-compassion can reduce these biased perceptions by helping individuals understand that their feelings are a natural part of human life and by fostering a connection with the broader human community (11, 67). Research has shown that the association between self-compassion and suicidal ideation is partially mediated by thwarted belongingness and perceived burdensomeness (68). Furthermore, high levels of self-compassion encourage individuals to adopt a more balanced and accepting stance toward suffering rather than over-identify with it (69), thereby reducing the likelihood of choosing suicide as an escape.

The results of the present study also indicate an interaction between mindfulness and perceived social support in predicting suicidal ideation. Specifically, individuals with higher perceived social support reported less suicidal ideation than those with lower perceived social support at the same level of mindfulness. Furthermore, perceived social support buffered the relationship between lower mindfulness and higher suicidal ideation, such that this association was attenuated at higher levels of perceived social support. This finding aligns with previous opinions suggesting that suicide results from a combination of internal factors and external social factors (36). Generally, adolescents with lower levels of mindfulness are more likely to develop suicidal ideation; however, if these adolescents perceive high levels of social support, their suicidal ideation tends to be relatively low. By testing the interaction between mindfulness and perceived social support, the current study goes beyond main effects to identify conditional processes: the protective association of mindfulness with suicidal ideation is not uniform across all adolescents but appears stronger among those reporting lower perceived social support. This insight addresses the study’s central aim of clarifying mechanisms linking mindfulness and social support to suicidal ideation, demonstrating that the effect of internal coping resources (mindfulness) can depend on external contextual resources (social support). Practically, this suggests that mindfulness-based interventions may be particularly beneficial for adolescents who lack sufficient perceived social support, while those with higher social support might benefit from interventions that strengthen their social networks.

The synergistic interplay among these variables is particularly informative within the Chinese context, a society traditionally characterized by its emphasis on interdependence and collective harmony. The data indicates that self-compassion functions as a key mechanism linking an individual’s internal state (mindfulness) with their external environment (social support). This finding challenges the assumption that mindfulness and social support are merely co-occurring protective factors and instead proposes a model where they work in concert to buffer against psychological distress. For adolescents in China, who navigate a demanding academic environment with high social expectations, the ability to be kind to oneself, recognize shared human suffering, and maintain a balanced perspective is essential.

This study’s findings have clear practical and policy implications. They suggest that school-based prevention efforts should address both intrapersonal capacities (e.g., mindfulness, self-compassion) and interpersonal support systems (e.g., family, peers, teachers) in an integrated manner. In the Chinese school context, interventions that simultaneously cultivate students’ capacity for mindful awareness and self-compassion while strengthening their perceived availability of supportive relationships are likely to be most effective in reducing suicidal ideation. Concretely, we recommend a school-centered approach that combines universal, selective, and indicated interventions. On the one hand, schools should promote mindfulness and self-compassion education for all students—for example, by incorporating brief mindfulness practices and self-compassion training into health education or curriculums, and by establishing small-group “mindfulness support groups” or “self-compassion groups” to enhance students’ emotional regulation and self-acceptance. On the other hand, schools should develop and institutionalize school-based social support systems—including teacher training in psychoeducation, peer support groups, and parent workshops that foster home–school collaboration—in order to strengthen students’ perceived support from teachers and parents.

This study has several limitations. First, due to the cross-sectional design, causal inferences cannot be drawn.; longitudinal research in the future is needed to determine temporal and causal relationships. Second, suicidal ideation was measured using a single item from the BDI; future studies would benefit from employing more comprehensive measures of suicidal ideation (e,g. the Beck Suicidal Ideation Scale). Third, all measures were collected via self-report questionnaires, which are vulnerable to social desirability bias. Incorporating additional data sources—such as behavioral observations, peer or caregiver reports, or physiological indicators—in future studies could help mitigate such biases and strengthen measurement validity. Finally, the use of convenience sampling may have resulted systematic biases with respect to geographic distribution and educational background, thereby limiting the generalizability of our findings. Future studies could employ stratified random sampling to improve representativeness and external validity.

## 5 Conclusion

In summary, this study, with a population of Chinese adolescents, confirms that mindfulness and perceived social support are related to the decrease of suicidal ideation and further illuminates the mediating role of self-compassion in these relationships. Higher levels of mindfulness and perceived social support may promote adolescents’ self-compassion, thereby reducing suicidal ideation. These findings contribute to a deeper understanding of the common mechanisms by which mindfulness and social support influence suicidal ideation, and have implications for developing interventions that enhance self-compassion in suicidal adolescents. For example, Mindfulness-based supportive group interventions and Compassionate focus therapy may be effective in alleviating suicidal ideation in adolescents.

## Data Availability

The raw data supporting the conclusions of this article will be made available by the authors, without undue reservation.

## References

[1] World Health Organization. Suicide worldwide in 2019 (2021). Available online at: https://www.who.int/publications/i/item/9789240026643 (Accessed June 13, 2024).

[2] Wiglesworth A. Risk for suicide in adolescence: how identity, development, and minority stress may shape neurobiological responses to social rejection. Biol Psychiatry. (2024) 95:1060–2. doi: 10.1016/j.biopsych.2024.04.007, PMID: 38811075

[3] Miron O Yu K-H Wilf-Miron R Kohane IS. Suicide rates among adolescents and young adults in the United States, 2000-2017. JAMA. (2019) 321:2362. doi: 10.1001/jama.2019.5054, PMID: 31211337 PMC6582264

[4] Zou G Lv J Qiao X. A meta-analysis of detection rate of suicidal ideation in middle school students in China. Chin Ment Health J. (2021) 35:643–50. doi: 10.3969/j.issn.1000-6729.2021.08.006

[5] O’Connor RC Kirtley OJ. The integrated motivational–volitional model of suicidal behaviour. Phil Trans R Soc B. (2018) 373:20170268. doi: 10.1098/rstb.2017.0268, PMID: 30012735 PMC6053985

[6] O’Neill C Pratt D Kilshaw M Ward K Kelly J Haddock G. The relationship between self-criticism and suicide probability. Clin Psychol Psychother. (2021) 28:1445–56. doi: 10.1002/cpp.2593, PMID: 33847028

[7] Neff KD. Self-compassion: an alternative conceptualization of a healthy attitude toward oneself. Self Identity. (2003) 2:85–101. doi: 10.1080/15298860309032

[8] Neff KD. The development and validation of a scale to measure self-compassion. Self Identity. (2003) 2:223–50. doi: 10.1080/15298860309027, PMID: 26979311

[9] Voci A Veneziani CA Fuochi G. Relating mindfulness, heartfulness, and psychological well-being: the role of self-compassion and gratitude. Mindfulness. (2019) 10:339–51. doi: 10.1007/s12671-018-0978-0

[10] Cleare S Gumley A O’Connor RC. Self-compassion, self-forgiveness, suicidal ideation, and self-harm: A systematic review. Clin Psychol Psychother. (2019) 26:511–30. doi: 10.1002/cpp.2372, PMID: 31046164

[11] Suh H Jeong J. Association of self-compassion with suicidal thoughts and behaviors and non-suicidal self injury: A meta-analysis. Front Psychol. (2021) 12:633482. doi: 10.3389/fpsyg.2021.633482, PMID: 34122224 PMC8192964

[12] Huang M Hou J. Childhood maltreatment and suicide risk: The mediating role of self-compassion, mentalization, depression. J Affect Disord. (2023) 341:52–61. doi: 10.1016/j.jad.2023.08.112, PMID: 37633526

[13] Bryan CJ Graham E Roberge E. Living a life worth living: Spirituality and suicide risk in military personnel. Spiritual Clin Pract. (2015) 2:74–8. doi: 10.1037/scp0000056

[14] Tesh M Learman J Pulliam RM. Mindful self-compassion strategies for survivors of intimate partner abuse. Mindfulness. (2015) 6:192–201. doi: 10.1007/s12671-013-0244-4

[15] Zeller M Yuval K Nitzan-Assayag Y Bernstein A. Self-compassion in recovery following potentially traumatic stress: longitudinal study of at-risk youth. J Abnorm Child Psychol. (2015) 43:645–53. doi: 10.1007/s10802-014-9937-y, PMID: 25234347

[16] Liu G Hao G Das N Ranatunga J Schneider C Yang L. Self-compassion, self-referential caudate circuitry, and adolescent suicide ideation. Transl Psychiatry. (2024) 14:334. doi: 10.1038/s41398-024-03037-0, PMID: 39164232 PMC11335956

[17] Bluth K Bryce A Lathren CR Park J Pflum S Clayton M. Reducing suicide ideation in transgender adolescents with mindful self-compassion: an open trial. Mindfulness. (2024) 15:3107–28. doi: 10.1007/s12671-024-02421-7

[18] Scheibner H Daniels A Guendelman S Utz F Bermpohl F. Self-compassion mediates the relationship between mindfulness and borderline personality disorder symptoms. J Pers Disord. (2017) 32:1–19. doi: 10.1521/pedi_2017_31_331, PMID: 29120280

[19] Masoumi S Amiri M Yousefi Afrashteh M. Self-compassion: the factor that explains a relationship between perceived social support and emotional self-regulation in psychological well-being of breast cancer survivors. Iran J Psychiatry. (2022) 17:341–9. doi: 10.18502/ijps.v17i3.9734, PMID: 36474692 PMC9699804

[20] Brown KW Ryan RM. The benefits of being present: Mindfulness and its role in psychological well-being. J Pers Soc Psychol. (2003) 84:822–48. doi: 10.1037/0022-3514.84.4.822, PMID: 12703651

[21] Kabat-Zinn J. Wherever You Go, There You Are: Mindfulness Meditation in Everyday Life. New York: Hachette Books (1994). 132 p.

[22] Neff KD Dahm KA. Self-compassion: what it is, what it does, and how it relates to mindfulness. In: Ostafin BD Robinson MD Meier BP, editors. Handbook of Mindfulness and Self-Regulation. Springer New York, New York, NY (2015). p. 121–37. doi: 10.1007/978-1-4939-2263-5_10

[23] Evans S Wyka K Blaha KT Allen ES. Self-compassion mediates improvement in well-being in a mindfulness-based stress reduction program in a community-based sample. Mindfulness. (2018) 9:1280–7. doi: 10.1007/s12671-017-0872-1

[24] Joss D Khan A Lazar SW Teicher MH. Effects of a mindfulness-based intervention on self-compassion and psychological health among young adults with a history of childhood maltreatment. Front Psychol. (2019) 10:2373. doi: 10.3389/fpsyg.2019.02373, PMID: 31749731 PMC6843003

[25] Bergen-Cico D Cheon S. The mediating effects of mindfulness and self-compassion on trait anxiety. Mindfulness. (2014) 5:505–19. doi: 10.1007/s12671-013-0205-y

[26] Yousefi Afrashteh M Hasani F. Mindfulness and psychological well-being in adolescents: the mediating role of self-compassion, emotional dysregulation and cognitive flexibility. Borderline Pers Disord Emotion Dysregul. (2022) 9:22. doi: 10.1186/s40479-022-00192-y, PMID: 36059027 PMC9441221

[27] Gu J Strauss C Bond R Cavanagh K. How do mindfulness-based cognitive therapy and mindfulness-based stress reduction improve mental health and wellbeing? A systematic review and meta-analysis of mediation studies. Clin Psychol Rev. (2015) 37:1–12. doi: 10.1016/j.cpr.2015.01.006, PMID: 25689576

[28] Sevel LS Finn MTM Smith RM Ryden AM McKernan LC. Self-compassion in mindfulness-based stress reduction: An examination of prediction and mediation of intervention effects. Stress Health. (2020) 36:88–96. doi: 10.1002/smi.2917, PMID: 31874122 PMC9281130

[29] Taylor SE. Social support: A review. In: Friedman HS, editor. The Oxford Handbook of Health Psychology. Oxford: Oxford University Press (2011). p. 190–214. doi: 10.1093/oxfordhb/9780195342819.013.0009

[30] Feeney BC Collins NL. A new look at social support: A theoretical perspective on thriving through relationships. Pers Soc Psychol Rev. (2015) 19:113–47. doi: 10.1177/1088868314544222, PMID: 25125368 PMC5480897

[31] Maheux A Price M. The indirect effect of social support on post-trauma psychopathology via self-compassion. Pers Individ Dif. (2016) 88:102–7. doi: 10.1016/j.paid.2015.08.051

[32] Zhou L Sukpasjaroen K Wu Y Gao L Chankoson T Cai E. Perceived social support promotes nursing students’ Psychological wellbeing: explained with self-compassion and professional self-concept. Front Psychol. (2022) 13:835134. doi: 10.3389/fpsyg.2022.835134, PMID: 35478770 PMC9037285

[33] Chu X Geng Y Zhang R Guo W. Perceived social support and life satisfaction in infertile women undergoing treatment: A moderated mediation model. Front Psychol. (2021) 12:651612. doi: 10.3389/fpsyg.2021.651612, PMID: 34122236 PMC8194393

[34] Toplu E Kemer G Pope A Moe J. Self-compassion matters: The relationships between perceived social support, self-compassion, and subjective well-being among LGB individuals in Turkey. J Couns Psychol. (2018) 65:372–82. doi: 10.1037/cou0000261, PMID: 29672086

[35] Wilson JM Weiss A Shook NJ. Mindfulness, self-compassion, and savoring: Factors that explain the relation between perceived social support and well-being. Pers Individ Dif. (2020) 152:109568. doi: 10.1016/j.paid.2019.109568

[36] Till B Arendt F Kirchner S Naderer B Niederkrotenthaler T. The role of monocausal versus multicausal explanations of suicide in suicide reporting: A randomized controlled trial. Suicide Life Threat Behav. (2023) 53:1063–75. doi: 10.1111/sltb.13007, PMID: 37823595

[37] De Aguiar KR Bilhalva JB Cabelleira MD Guimarães GO Madureira T Agako A. The impact of mindfulness on suicidal behavior: a systematic review. Trends Psychiatry Psychother. (2022) 44:e20210316. doi: 10.47626/2237-6089-2021-0316, PMID: 34551465 PMC9907375

[38] De Jaegere E Dumon E van Heeringen K van Landschoot R Stas P Portzky G. Mindfulness-based cognitive therapy for individuals who are suicidal: A randomized controlled trial. Arch Suicide Res. (2024) 28(4):1228–48. doi: 10.1080/13811118.2023.2282663, PMID: 37994872

[39] Liang X Chang W Ran H Fang D Che Y Wang S. Childhood maltreatment and suicidal ideation in Chinese children and adolescents: the mediating role of mindfulness. BMC Psychiatry. (2022) 22:680. doi: 10.1186/s12888-022-04336-w, PMID: 36333697 PMC9635069

[40] Liu H Wang W Qi Y Zhang L. Suicidal ideation among Chinese survivors of childhood sexual abuse: Associations with rumination and perceived social support. Child Abuse Negl. (2022) 123:105420. doi: 10.1016/j.chiabu.2021.105420, PMID: 34902640

[41] Wan L Yang X Liu B Zhang Y Liu X Jia C. Depressive symptoms as a mediator between perceived social support and suicidal ideation among Chinese adolescents. J Affect Disord. (2022) 302:234–40. doi: 10.1016/j.jad.2022.01.061, PMID: 35090945

[42] Chen S Cui H Zhou R Jia Y. Revision of mindful attention awareness scale (MAAS). Chin J Clin Psychol. (2012) 20:148–51. doi: 10.16128/j.cnki.1005-3611.2012.02.024

[43] Jiang Q. Perceived social support scale. In: Wang X Wang X Ma H, editors. Rating Scales for Mental Health. Beijing: Chinese Mental Health Journal Publisher (1999). p. 131–3.

[44] Zimet GD Dahlem NW Zimet SG Farley GK. The multidimensional scale of perceived social support. J Pers Assess. (1988) 52:30–41. doi: 10.1207/s15327752jpa5201_2, PMID: 2280326

[45] Ye J. Perceived social support, enacted social support and depression in a sample of college students. J Psychol Sci. (2006) 29:1141–3. doi: 10.16719/j.cnki.1671-6981.2006.05.027

[46] Gong H Jia H Guo T Zou L. The revision of self-compassion scale and its reliability and validity in adolescents. Psychol Res. (2014) 7:36–40.

[47] Beck AT Steer RA Brown G. Manual for the Beck Depression Inventory-II. San Antonio: TX: Psychological Corporation (1996).

[48] Hu L Bentler PM. Cutoff criteria for fit indexes in covariance structure analysis: Conventional criteria versus new alternatives. Struct Equ Modeling. (1999) 6:1–55. doi: 10.1080/10705519909540118

[49] Duan J Xu Y Yu L. Theory of self-cultivation extracted from Confucianism: A reflection and supplement to social exchange paradigm. Adv Psychol Sci. (2018) 26:1890–900. doi: 10.3724/SP.J.1042.2018.01890

[50] Xu L Zhan G. Social self-criticism and the shaping of Chinese national identity. J Knowl Econ. (2025) 16:5385–418. doi: 10.1007/s13132-024-02122-5

[51] Neff KD Pisitsungkagarn K Hsieh Y-P. Self-compassion and self-construal in the United States, Thailand, and Taiwan. J Cross-Cult Psychol. (2008) 39:267–85. doi: 10.1177/0022022108314544

[52] Huang S Kan PF. Chinese American immigrant parents’ Socialization of emotions in bilingual bicultural preschool children. Front Psychol. (2021) 12:642417. doi: 10.3389/fpsyg.2021.642417, PMID: 34393881 PMC8362853

[53] Fang J Brown GTL Hamilton R. Changes in Chinese students’ academic emotions after examinations: Pride in success, shame in failure, and self-loathing in comparison. Br J Educ Psychol. (2023) 93:245–61. doi: 10.1111/bjep.12552, PMID: 36239121 PMC10091958

[54] Liu Q-Q Hu Y-T. Self-compassion mediates and moderates the association between harsh parenting and depressive symptoms in Chinese adolescent. Curr Psychol. (2023) 42:16036–48. doi: 10.1007/s12144-020-01034-2

[55] Liu Y Xiao Y Ran H He X Jiang L Wang T. Association between parenting and non-suicidal self-injury among adolescents in Yunnan, China: a cross-sectional survey. PeerJ. (2020) 8:e10493. doi: 10.7717/peerj.10493, PMID: 33354431 PMC7727394

[56] Moscardini EH Robinson A Gerner J Tucker RP. Perceived stress and suicidal ideation: The role of dispositional mindfulness. Suicide Life Threat Behav. (2023) 53:776–86. doi: 10.1111/sltb.12982, PMID: 37530498

[57] Baumeister RF. Suicide as escape from self. Psychol Rev. (1990) 97:90–113. doi: 10.1037/0033-295X.97.1.90, PMID: 2408091

[58] Schmelefske E Per M Khoury B Heath N. The effects of mindfulness-based interventions on suicide outcomes: A meta-analysis. Arch Suicide Res. (2022) 26:447–64. doi: 10.1080/13811118.2020.1833796, PMID: 33126844

[59] Joiner T. Why People Die by Suicide. Cambridge: Harvard University Press (2005). 304 p.

[60] Arenson M Bernat E De Los Reyes A Neylan TC Cohen BE. Social support, social network size, and suicidal ideation: A nine-year longitudinal analysis from the Mind Your Heart Study. J Psychiatr Res. (2021) 135:318–24. doi: 10.1016/j.jpsychires.2021.01.017, PMID: 33545566

[61] Chu C Buchman-Schmitt JM Stanley IH Hom MA Tucker RP Hagan CR. The interpersonal theory of suicide: A systematic review and meta-analysis of a decade of cross-national research. Psychol Bull. (2017) 143:1313–45. doi: 10.1037/bul0000123, PMID: 29072480 PMC5730496

[62] Kabat-Zinn J. Coming to Our Senses: Healing Ourselves and the World Through Mindfulness. London: Hachette UK (2005). 732 p.

[63] Aydin Sünbül Z Yerin Güneri O. The relationship between mindfulness and resilience: The mediating role of self-compassion and emotion regulation in a sample of underprivileged Turkish adolescents. Pers Individ Dif. (2019) 139:337–42. doi: 10.1016/j.paid.2018.12.009

[64] Germer C. The Mindful Path to Self-Compassion: Freeing Yourself from Destructive Thoughts and Emotions. New York: Guilford Press (2009). 321 p.

[65] Jeon H Lee K Kwon S. Investigation of the structural relationships between social support, self-compassion, and subjective well-being in Korean elite student athletes. Psychol Rep. (2016) 119:39–54. doi: 10.1177/0033294116658226, PMID: 27381414

[66] Neff KD McGehee P. Self-compassion and psychological resilience among adolescents and young adults. Self Identity. (2010) 9:225–40. doi: 10.1080/15298860902979307

[67] Rabon JK Hirsch JK Kaniuka AR Sirois F Brooks BD Neff K. Self-compassion and suicide risk in veterans: when the going gets tough, do the tough benefit more from self-compassion? Mindfulness. (2019) 10:2544–54. doi: 10.1007/s12671-019-01221-8

[68] Umphrey LR Sherblom JC Swiatkowski P. Relationship of self-compassion, hope, and emotional control to perceived burdensomeness, thwarted belongingness, and suicidal ideation. Crisis. (2021) 42:121–7. doi: 10.1027/0227-5910/a000697, PMID: 32672522

[69] Liu A Wang W Wu X. Self-compassion and posttraumatic growth mediate the relations between social support, prosocial behavior, and antisocial behavior among adolescents after the Ya’an earthquake. Eur J Psychotraumatol. (2021) 12:1864949. doi: 10.1080/20008198.2020.1864949, PMID: 34025914 PMC8128115

